# Roles of Copper Transport Systems Members in Breast Cancer

**DOI:** 10.1002/cam4.70498

**Published:** 2024-12-15

**Authors:** Yichang Chen, Chen Li, Mengxin Li, Bing Han

**Affiliations:** ^1^ Department of Breast Surgery, General Surgery Center First Hospital of Jilin University Changchun China; ^2^ Department of Neurosurgery First Hospital of Jilin University Changchun China

**Keywords:** ATP7A and ATP7B, breast cancer, copper, copper transport systems, SLC31A1

## Abstract

**Background:**

The occurrence and progression of breast cancer are closely linked to copper ion homeostasis. Both copper deficiency and excess can inhibit breast cancer growth, while copper transport systems may contribute to its progression by regulating copper ion transport and the activity of associated proteins. However, a comprehensive review of the roles and applications of copper transport systems in breast cancer remains limited. In this study, we summarize the workflow of copper transport systems and the dual role of copper in cancer, highlighting the contributions of specific members of the copper transport system to breast cancer.

**Methods:**

A comprehensive search of the PubMed database was conducted to identify articles published over the past 30 years that focus on the relationship between copper transport system members and breast cancer. The findings were synthesized to elucidate the roles and mechanisms of these transporters in the onset and progression of breast cancer.

**Results:**

We identified 13 members of the copper transport system associated with the occurrence, progression, and mortality of breast cancer, including SLC31A1, DMT1, ATP7A, ATP7B, MTs, GSH, ATOX1, CCS, COX17, SCO1, SCO2, and COX11. Our findings revealed that, apart from STEAP, the remaining 12 members were overexpressed in breast cancer. These members influence the onset, progression, and cell death of breast cancer by modulating biological pathways such as intracellular copper ion levels and ROS. Notably, we observed for the first time that depletion of the copper storage protein GSH leads to increased copper ion accumulation, resulting in cuproptosis in breast cancer cells.

**Conclusion:**

By integrating the members of the copper transport system in breast cancer, we offer novel insights for the treatment of breast cancer and copper‐related therapies.

**Abbreviations**
ATOX1: antioxidant 1
ATP7A: ATPase copper transporting alpha
ATP7B: ATPase copper transporting beta gene
CCO: cytochrome c oxidase
CCS: superoxide dismutase
COX: cytochrome c oxidase
CPT: camptothecin
Cu: copper
DFO: deferoxamine
DMT1: divalent metal transporter 1
DOX: doxorubicin
ECM: extracellular matrix
EMT: epithelial–mesenchymal transition
eNOS: endothelial nitric oxide synthase
ER: estrogen receptors
GRX1: glutaredoxin1
GSH: glutathione
HBXIP: hepatitis B X‐interacting protein
JNK: c‐Jun N‐terminal kinase
LOX: lysyl oxidase
MBDs: metal‐binding domains
MTCO1/2, COX1/2: mitochondrially encoded cytochrome c oxidase l/II
MTs: metallothioneins
OXPHOS: oxidative phosphorylation
PR: progesterone receptors
SLC31A1: copper transporter 1
SOD: superoxide dismutase
TCA: tricarboxylic acid
TM: tetrathiomolybdate
TNBC: triple‐negative breast cancer
TRIEN: triethylenetetramine

## 1 Introduction

Copper is one of the indispensable micronutrients in the occurrence and development of breast cancer, and maintaining copper homeostasis is very important for breast cancer. Copper transport systems effectively supply adequate copper for essential physiological processes while concurrently preventing the accumulation of copper to mitigate potential tissue toxicity. The regulation of the copper transport systems is accomplished through the precise coordination of multiple transporters and chaperone proteins, including copper influx transporters (SLC31A1 and DMT1), copper efflux transporters (ATP7A and ATP7B), copper storage protein (MTs, GSH), and copper chaperone proteins (ATOX1, CCS, COX17, SCO1, SCO2, and COX11) [1, 2, 3].

As far as cancer cell survival is concerned, copper has both stimulatory and inhibitory functions [1, 4]. Copper can promote tumor vascular growth, invasion, and metastasis, regulate mitochondrial function, and participate in a variety of signal transduction [1, 5]. Copper can also participate in the programmed death of tumor cells, such as autophagy, ferroptosis, and cuproptosis [4, 6, 7]. Members of the copper transport system also influence the occurrence, development, invasion, metastasis, and chemotherapy resistance of breast cancer (Table 1).

**TABLE 1:** The roles of copper transport systems members in breast cancer.

Member	Type	Roles in breast cancer
SLC31A1	Copper influx transporter	High expression; Associated with poor prognosis; SLC31A1 regulates immunotherapy and chemotherapy resistance in breast cancer; SLC31A1 promotes breast cancer development by activating the PI3K‐PDK1‐AKT signaling pathway and the EMT gene‐phenotype; SLC31A1 increases intracellular copper ions and ROS, inducing DNA damage and death of MDA‐MB‐231 [8, 9, 10, 11, 12, 13, 14].
DMT1	Copper influx transporter	High expression; Associated with poor prognosis; In iron‐deficient MDA‐MB‐231 cells or after inhibition by HO‐1 and activation by Hipo‐Yap pathway, DMT1 promotes migration and development of breast cancer cells by regulating iron metabolism; DMT1 upregulated by sulfasalazine and DOX contribute to the occurrence of ferroptosis in breast cancer cells [15, 16, 17, 18, 19].
STEAP	Copper reductase	Low expression; Associated with good prognosis; STEAP1 and STEAP2 inhibit the invasion, proliferation, and metastasis of breast cancer by inhibiting EMT [20, 21].
ATP7A	Copper efflux transporter	High expression; Associated with poor prognosis; ATP7A promotes the development and metastasis of breast cancer by maintaining and increasing LOX activity, regulating copper ion and ROS homeostasis, and increasing cisplatin resistance in breast cancer and helping breast cancer immune escape. Downregulation of ATP7A induces cuproptosis in breast cancer by promoting copper ion accumulation [22, 23, 24, 25, 26].
ATP7B	Copper efflux transporter	High expression; There was no significant difference in survival rate with breast cancer; ATP7B promotes the development of breast cancer by participating in cell cycle, oxidative phosphorylation, DNA replication pathways, and immune escape of breast cancer. Downregulation of ATP7B induces cuproptosis in breast cancer by promoting copper ion accumulation [23, 24, 26].
ATOX1	Copper chaperone	High expression; It is associated with poor prognosis in early breast cancer patients; ATOX1 transports copper and mediates breast cancer cell migration through the ATP7A‐LOX axis; ATOX1 maintains TNBC survival by promoting angiogenesis, avoiding excess copper and ROS accumulation and increasing paclitaxel resistance in breast cancer [27, 28, 29].
CCS	Copper chaperone	High expression; In MDA‐MB‐231 and MCF‐7, CCS promotes the growth and migration of breast cancer cells via regulating the ERK1/2 activity mediated by ROS [30].
COX17	Mitochondrial copper chaperone	High expression; Associated with poor prognosis; COX17 contributes to metastasis, recurrence, and tamoxifen resistance of breast cancer [31].
SCO1	Mitochondrial copper chaperone	SCO1 promotes the occurrence of breast cancer [32].
SCO2	Mitochondrial copper chaperone	SCO2 regulates metabolic pathways in breast cancer [33, 34].
COX11	Mitochondrial copper chaperone	Associated with poor prognosis [35, 36].
MTs	Copper storage protein	Associated with poor prognosis; MTs promote the proliferation of breast cancer cells by promoting the cell cycle, regulating oxidative stress, inhibiting apoptosis, increasing DOX resistance of breast cancer, and enhancing the aggressiveness by upregulating MMP‐9 via AP‐1 and NF‐κB activation [37, 38, 39, 40, 41].
GSH	Copper storage protein	GSH regulates the oxidative stress of breast cancer cells and stores copper ions; copper overload caused by GSH consumption leads to lipoylated protein aggregation and iron–sulfur protein loss, which ultimately leads to cuproptosis [42, 43].

Both copper deficiency and copper overload are promising therapies in cancer therapy. In addition to the classical copper chelating agent tetrathiomolybdate (TM), which can be used to treat breast cancer by regulating copper homeostasis [44], the newly discovered copper chelating agent hydroxytyrosol forms a complex with copper to regulate intracellular copper levels by downregulating the phosphorylation of copper‐dependent AKT, and inhibits the aggressivity of triple‐negative breast cancer (TNBC) [45]. The copper chelating agent triethylenetetramine (TRIEN) reduces bioavailability of Cu in breast cancer cells, thereby inhibiting AKT‐driven EMT (epithelial–mesenchymal transition) activation [46]. Cuproptosis is a recently identified form of programmed cell death that induces the aggregation of mitochondrial lipoylated proteins and the destabilization of Fe‐S cluster proteins due to intracellular copper accumulation [4]. The currently popular copper ionophores, elesclomol and disulfiram, can induce cuproptosis in tumor cells [4, 47]. A cuproptosis‐based nanomedicine, Type‐I AIE photosensitizer‐loaded biomimetic system (PTC) has been shown to significantly inhibit breast cancer formation and metastasis by inducing cuproptosis [42]. Pyrithione zinc (ZnPT) is a compound that significantly inhibits the progression of TNBC, and it has been found that ZnPT can induce cuproptosis by disrupting copper homeostasis and DLAT oligomerization [48].

Copper ions are crucial in regulating the growth and death of breast cancer, so copper‐related anticancer therapy is of great significance. This paper aims to summarize the association between members of the copper transport systems and breast cancer, and lay a foundation for our future research.

## 2 Copper Transport Systems—The Core Force That Maintains Copper Homeostasis

**FIGURE 1:**
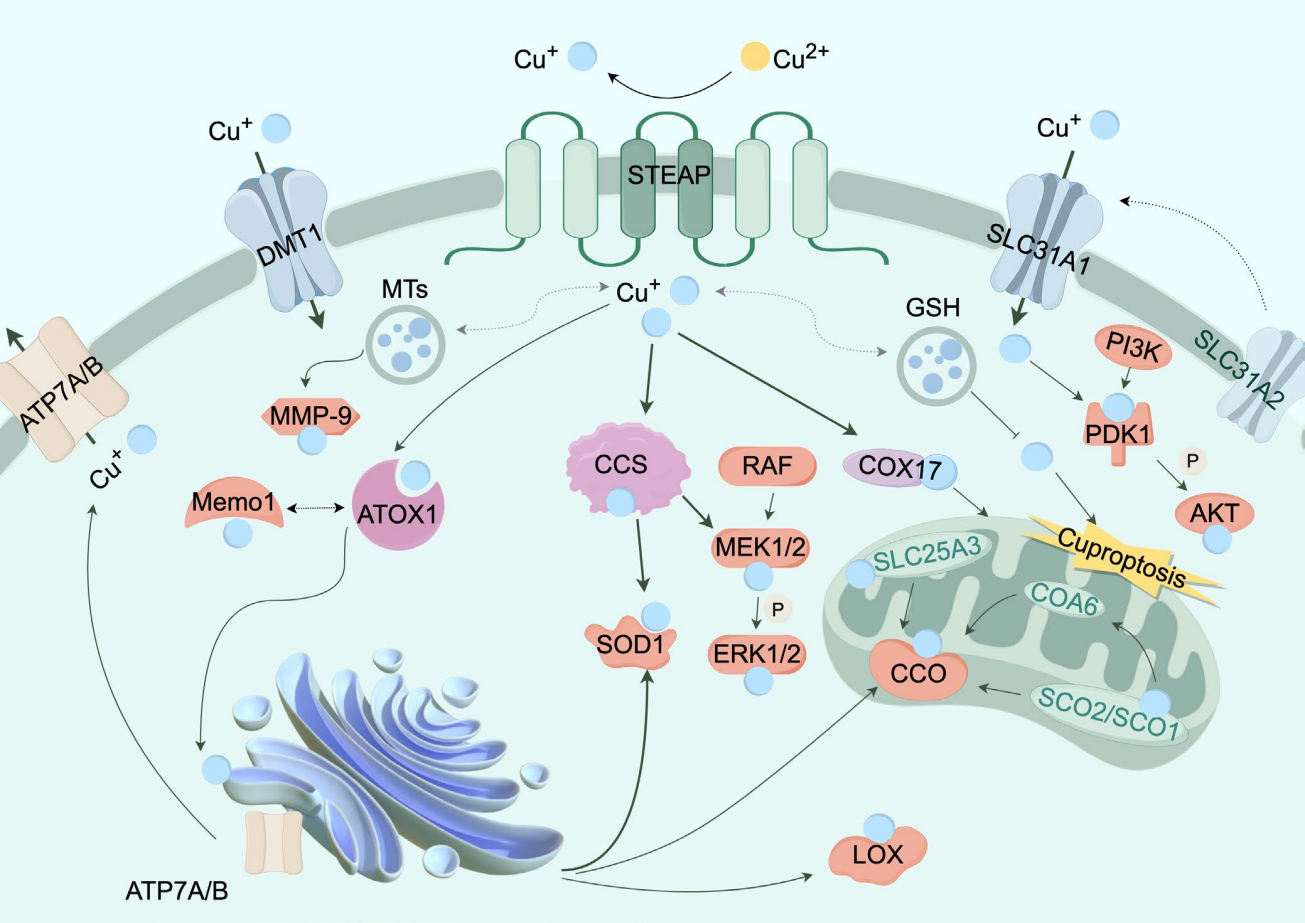
Simplified overview of copper transport systems: SLC31A1 and DMT1 are responsible for the uptake of Cu(I), and The reduction of Cu(II) to Cu(I) is attributed to the activity of the STEAP. SLC31A2 potentially exerts a regulatory function in the Cu‐dependent mobilization of SLC31A1. Ingested Cu(I) is transmitted by copper chaperones ATOX1, CCS, and COX17. MTs and GSH are responsible for storing Cu(I). ATP7A and ATP7B are responsible for copper output. In mitochondria, SCO2 and COA6 jointly assist SCO1 to maintain REDOX balance and promote SCO1 to bind Cu(I) and transfer to CCO. The mitochondrial transporter SLC25A3 binds and transports Cu(I), ultimately aiding in the metallization of CCO. These copper transporters work together to ensure the metallization of copper‐dependent proteins, including CCO, LOX, SOD1, etc.

Copper is a vital micronutrient that exhibits redox properties, thereby exerting both advantageous and detrimental effects on cellular processes. In mammalian cells, it is generally observed that copper is transported in the form of Cu(I). In humans, it is distributed throughout the body and participates in a range of physiological processes [1, 49]. Copper transport systems make a great contribution to maintaining copper homeostasis. We often divide the copper transport systems into four main parts, which are responsible for the input, output, transport, and storage of copper (Figure 1).

### 2.1 Copper Input

**FIGURE 2:**
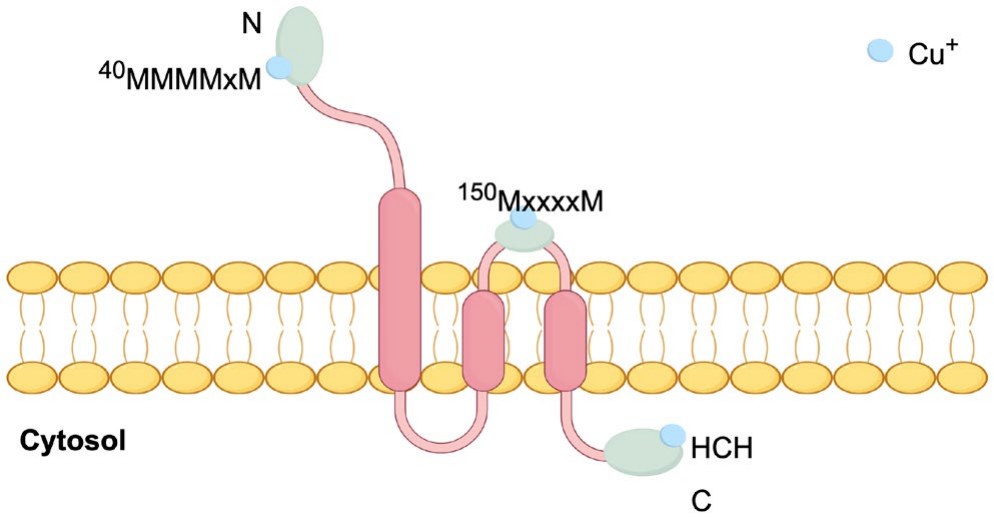
SLC31A1 consists of three transmembrane domains, with the N‐terminal located outside the cell and the C‐terminal located inside the cytoplasm. The possible binding sites of copper include ^40^MMMMxM in the N‐terminal extracellular domain (where M represents methionine and x represents a variable amino acid), ^150^MxxxxM in the second transmembrane domain, and ^189^HCH in the cytoplasmic C‐terminal domain.

SLC31A1 (CTR1) is considered the principal constituent of the CTR family, which encompasses a total of six identified members, namely CTR1–6 [50]. CTR1, characterized by its high affinity for copper, is accountable for approximately 70% Cu uptake in human cells [51]. This trimeric homo‐protein undergoes glycosylation at both N‐ and O‐sites. It is primarily localized at the plasma membrane [52]. The extracellular amino terminus of human CTR1 contains Cu‐binding amino acids, including two clusters of methionine and histidine, as well as neighboring aspartates. These specific amino acids are responsible for binding the Cu(I) and Cu(II) forms of copper before it enters the cell. It is believed that this binding process helps direct copper towards the transmembrane channel, which is located in the center of the trimer structure [53, 54, 55]. The main copper binding sites include ^40^MMMMxM in the N‐terminal extracellular domain (where M represents methionine and x represents a variable amino acid), ^150^MxxxxM in the second transmembrane domain, and ^189^HCH in the cytoplasmic C‐terminal domain [55] (Figure 2). CTR2 is a structural homolog of CTR1, characterized by the absence of the Cu‐binding domain [56]. There has been a proposal indicating that CTR2 facilitates the external domain cutting of CTR1, suggesting its potential involvement in regulating the Cu‐dependent mobilization of CTR1. Nevertheless, CTR2 lacks significant Cu transport activity when functioning independently and undergoes degradation in the absence of CTR1. The divalent metal transporter 1 (DMT1) is capable of the transport of copper in select cellular environments and under specific conditions [2]. The application of DMT1 antisense oligonucleotide to intestinal Caco‐2 cells results in a notable decrease in copper absorption [57]. Multiple studies have provided evidence that DMT1 is capable of facilitating the importation of copper in cases where there is a deficiency of CTR1. Simultaneous gene silencing of CTR1 and DMT1 results in complete suppression of copper uptake [57]. CTR1 and DMT1 would compensate for each other for copper uptake in mammalian cells. The Six‐Transmembrane Epithelial Antigen of the Prostate (STEAP) protein family has a copper reduction function, which can decrease Cu(II) to Cu(I) and may promote copper uptake by CTR1 [1].

### 2.2 Copper Output

**FIGURE 3:**
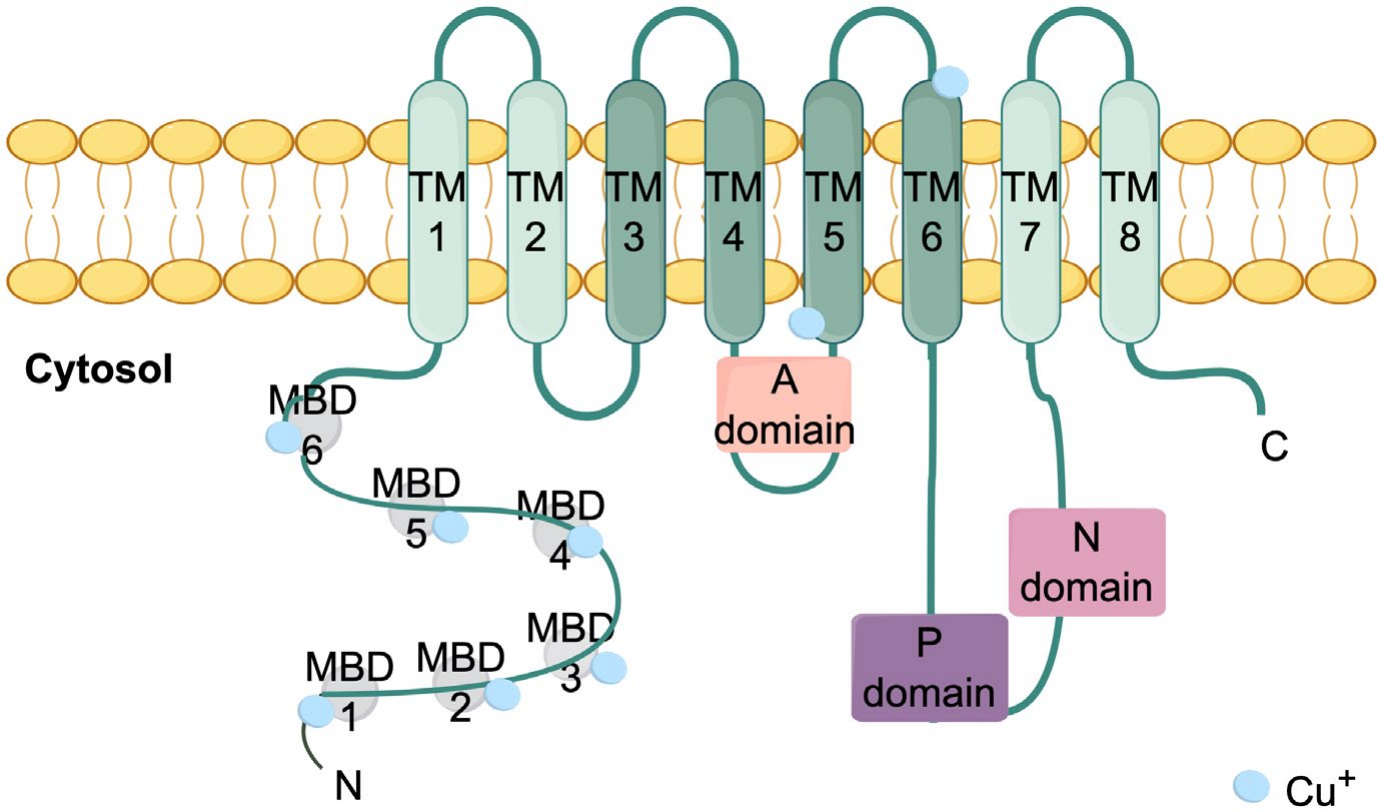
ATP7A and ATP7B each have 8 transmembrane domains (TMD) and 6N‐terminal metal binding domains (MBDs), and each metal binding domain has a CXXC motif to facilitate copper binding. In addition, there are also Cu binding sites in TM5 and TM6.

Mutations in the P‐type ATPase transporters, namely ATP7A (ATPase copper transporting alpha) and ATP7B (ATPase copper transporting beta Gene), have been identified as the underlying cause of Menkes and Wilson diseases [58], respectively, and regulate cytoplasmic copper levels through the active transportation of copper ions out of cells or into the endomembrane system [59, 60, 61]. The ATP7A/B encompasses a P‐type ATPase core that comprises a membrane transport domain and three cytoplasmic domains, namely the A, P, and N domains [61] (Figure 3). Additionally, it possesses a distinctive amino terminus that consists of six consecutive metal‐binding domains (MBDs) [62]. The ferredoxin‐like fold, which is conserved across different domains, encompasses approximately 70 amino acids and is characterized by the presence of the invariant CxxC motif, which plays a crucial role in binding Cu(I) [60, 61, 63]. In most cells and tissues, ATP7A typically exhibits higher levels of abundance compared to ATP7B and assumes a central role in the transportation of Cu to Cu‐dependent enzymes, as well as in the efflux of Cu [64, 65]. In contrast, ATP7B is expressed at much higher levels than ATP7A in the liver, kidneys, and placenta, and plays a crucial role in eliminating copper [61, 66]. The ATP7A/B exhibits localization to the Golgi apparatus under steady‐state conditions [67]. At the Golgi apparatus, ATP7A/B proteins facilitate the delivery of Cu to recently synthesized Cu‐dependent enzymes. These enzymes include cytochrome c oxidase (CCO, COX), superoxide dismutase (SOD1, SOD3), as well as various oxygenase and oxidase enzymes such as tyrosinase, lysyl oxidase (LOX), dopamine β‐hydroxylase, and copper amine oxidases. The presence of copper as a cofactor in these enzymes is crucial for their ability to catalyze a range of biologically significant biochemical reactions [1]. The translocation of ATP7A and ATP7B from the Golgi complex to the cell surface occurs through post‐Golgi vesicles in fibroblasts [68]. In hepatocytes, ATP7B is transported to the canalicular surface through the utilization of lysosomes [69].

### 2.3 Copper Transport

Copper ions that have been absorbed are conveyed to the secretory pathway and mitochondria through the assistance of copper chaperones, including ATOX1, CCS, and COX17 [2].

Antioxidant 1 (ATOX1), which is a chaperone for Cu, was previously referred to as the human ATX1 homolog (HAH1). It has been discovered that ATOX1 plays a crucial role in preserving the balance of cellular Cu homeostasis, mitigating oxidative stress, and regulating transcription [70]. Atox1 facilitates the transportation of copper to the secretory pathway, where it subsequently transfers copper to the copper‐transporting ATPases ATP7A and ATP7B. These ATPases are situated in the trans‐Golgi network as well as different endocytic vesicles [71]. It is postulated that the transfer of copper occurs from Atox1 to one or more of the MBDs, subsequently progressing to the copper‐binding site located within the transmembrane domain and ultimately reaching an acceptor on the opposing side of the membrane [60]. The available research indicates that Memo1 has the ability to bind to copper ions that have been reduced, as well as interact with the copper chaperone ATOX1 [72].

The genes known as copper chaperone for superoxide dismutase (CCS) are responsible for encoding the copper chaperone for cytosolic superoxide dismutase (SOD1). These genes have an impact on the activity of SOD1 by regulating the delivery of copper to SOD1 from its target [71]. SOD1 is an essential antioxidant enzyme responsible for eliminating reactive oxygen species within the cell by engaging in redox cycling facilitated by a catalytic copper ion, which is supplied by its copper chaperone (CCS) [73]. The process of CCS‐mediated metalation of SOD1 encompasses three discrete domains of CCS, all of which are essential for the proper functioning of SOD1. It is observed that Domain 1 (D1) obtains Cu(I) from the membrane transporter SLC31A1. Domain 2 (D2) imitates SOD1 to facilitate interactions between proteins. Additionally, Domain 3 (D3) is potentially engaged in the transfer of Cu from D1 to D3 while simultaneously generating the disulfide bond that is essential for SOD1 activation [74, 75]. Furthermore, the study found that CCS exhibited specific binding affinity towards MEK1 and played a crucial role in facilitating the transfer of copper to this protein. The researchers used mutants of CCS that interfere with the acquisition and exchange of Cu(I), as well as a small‐molecule inhibitor targeting CCS. The utilization of these tools led to a decrease in Cu‐stimulated MEK1 kinase activity [76].

The COX17, which serves as a Cu chaperone for CCO, has been found to play a role in the transport of copper ions from the cytosol to the mitochondria. This is attributed to its ability to localize in both the cytosol and the intermembrane space of the mitochondria [77, 78]. It delivers copper to additional chaperones, such as SCO1, SCO2, and COX11, within the mitochondria for synthesis and maturation of CCO [3, 78], which catalyzes the terminal reaction of the respiratory chain, which is located within the inner membrane of the mitochondria [71].

The mitochondrial Cu transporter PiC2 (SLC25A3) protein, which is situated in the inner membrane of mitochondria, binds Cu and plays a crucial role in the transportation of Cu ions and facilitates the metalation process of CCO in eukaryotic cells [79]. The involvement of COX19 in the maintenance of the redox state of COX11 and the facilitation of copper transportation to the COX1 subunit has been demonstrated [80]. Although there's not enough evidence that COA6 is a copper Chaperones, COA6 exerts its action on COX2 and SCO1 enzymes by facilitating the reduction of the disulfide bond present between their copper‐coordinating cysteine residues, thereby enabling the subsequent binding of copper ions [81]. Copper‐deposited ceria nanoparticles (CuCe NPs) are metal oxide nanomaterials that have stronger antioxidant properties by reducing the consumption of glutathione through the release of copper [82]. Studies have shown that the utilization of Ceria Nanoparticles as Copper Chaperones can effectively activate SOD1, thereby facilitating synergistic antioxidant therapy for the treatment of ischemic vascular diseases [82].

### 2.4 Copper Storage

Copper can be stored in glutathione (GSH) and metallothioneins (MTs). Cu(I)‐GSH transfers copper to MTs, ATOX1, and SOD1 and regulates the copper transport activity of SLC31A1 and ATP7A/B [2, 83, 84, 85]. Glutaredoxin1 (GRX1) functions as a thiol oxidoreductase that is dependent on GSH. The function of ATP7A/B depends on the activity of GRX1, which serves as a catalyst for either the reduction of intramolecular disulfide bonds or the deglutathionylation of cysteine residues within the CxxC motifs. These enzymatic processes are essential for copper binding and facilitate subsequent copper transport [86]. There are four main subfamilies of MTs (MT1‐4). MT1 and MT2 are expressed in all mammalian tissues, MT3 is mainly expressed in the nervous system, and MT4 is mainly expressed in the skin [87]. MT1 and MT2, which are two out of three isoforms of thiol‐rich proteins exhibiting a strong affinity for binding multiple copper ions, are considered to be the putative counterparts of the iron‐storing protein known as ferritin [1, 88].

## 3 The Copper in Cancer Is A Double‐Edged Sword

Copper has exhibited a significant correlation with multiple signaling pathways and tumor‐related biological behaviors. The progression of cancer is linked to elevated levels of cellular Cu concentrations. This association is observed in various cancer hallmarks, including proliferative immortality, angiogenesis, and metastasis, which have specific Cu‐related requirements [1]. Numerous studies in the field of cancer research have reported a notable elevation in the concentrations of copper ions within the tissues and serum of individuals afflicted with tumors as compared to those without such conditions [89]. Nevertheless, the toxicity of copper arises when concentrations surpass a threshold that is regulated by homeostatic mechanisms conserved throughout evolution [4].

Copper can facilitate the formation of blood vessels, which play a role in the initiation, growth, and spread of tumors. This is achieved through the activation of various proangiogenic factors, such as VEGF, FGF‐1, FGF‐2, TNF, and IL‐1, among others, either directly or indirectly. Additionally, new targets and pathways that rely on copper‐dependent signaling have been identified in this context [1, 5]. Furthermore, copper serves as a co‐factor for various members of the LOX enzyme family, which are responsible for facilitating the crosslinking process of collagen and elastin [90]. Consequently, copper plays a significant role in the stabilization of the extracellular matrix (ECM) [90], and the deprivation of copper has the ability to impede the epithelial‐to‐mesenchymal transition (EMT) of tumor cells [8]. Copper chaperone ATOX1 enhances LOX activity through the SLC31A1‐ATOX1‐ATP7A pathway and promotes VEGF‐induced endothelial cell angiogenesis [22, 91, 92]. In short, copper and copper transport systems play an important role in angiogenesis. Copper has been shown to increase the activity of endothelial nitric oxide synthase (eNOS) in a Ca^2+^‐dependent manner, thereby inducing vasodilation via the NO pathway [93, 94]. In addition, copper binds PDK1 and activates its downstream substrate AKT in a PI3K‐dependent manner to promote tumorigenesis [9].

Copper is involved in the regulation of neoplastic mitochondrial functions [1]. Studies have shown that copper deficiency can regulate mitochondrial oxidative phosphorylation (OXPHOS) and impair TNBC metastasis by reducing CCO activity, activating downstream of AMPK, and decreasing mTORC1 activity [44]. Different studies have also demonstrated that an overabundance of copper has the potential to impede the activation of S6K1 and its subsequent glycolytic‐related molecules, such as GLUT1, PKM2, and LDHA, consequently leading to the suppression of tumor growth [47]. The disruption of copper homeostasis not only affects mitochondrial respiration but also induces alterations in glycolysis and autophagy. Regulation of autophagy by copper and copper transport systems introduces novel copper‐dependent targets that have the potential to impact the growth and advancement of tumors. Copper can directly interact with MAPK 1/2 (MEK1/2) and ULK1/2 kinases and regulate their activity [6, 95], thereby promoting the phosphorylation of ATG13 and ERK1/2, triggering activation of the downstream c‐Jun N‐terminal kinase (JNK) pathway, leading to the formation of autophagy complexes, and ultimately promoting tumor growth [89, 96]. On the contrary, there are different findings that block copper absorption through SLC31A1 interference and copper chelator TM treatment could cause an observed augmentation in the process of autophagy as a means to counteract the mortality of pancreatic cancer cells [97].

In addition, there is a correlation between copper and ferroptosis. Some researchers have found that copper accumulation and activation of copper transport systems can promote ferroptosis by promoting the degradation of GPX4 or SLC7A11 [7, 98]. On the contrary, other scholars have found that ferroptosis can also be promoted in the case of copper depletion. The depletion of copper in dermal papilla cells, mediated by Bathocuproinedisulfonic, significantly intensifies the occurrence of ferroptosis. This effect is primarily attributed to a reduction in the levels of GSH and the inactivation of GPX4 [99]. The results of a recent study suggest that the accumulation of copper within cells serves as a trigger for the aggregation of lipoylated proteins associated with the tricarboxylic acid (TCA) cycle in mitochondria. Furthermore, this accumulation of copper also leads to the destabilization of Fe‐S cluster proteins. As a result, a distinct form of cell death, referred to as cuproptosis, is induced [4].

## 4 Copper Transport Systems and Breast Cancer

Copper concentrations in plasma/serum and tissue and expressions of most members of the copper transport systems are significantly greater in patients with breast cancer than in those without breast cancer and are strongly linked with unfavorable prognosis in breast cancer patients [10, 15, 27, 30, 31, 33, 58, 100, 101, 102]. The members of copper transport systems affect the occurrence, development, invasion, metastasis, and chemotherapy resistance of breast cancer in many ways (Table 1). Interfering with copper ions in breast cancer patients to fight cancer is a promising approach, so the study of copper transport systems and breast cancer is necessary.

### 4.1 SLC31A1

The star gene of cuproptosis, SLC31A1, is upregulated in breast cancer [10] and can act as a sentinel for poor prognosis in breast cancer patients [11, 12]. It was found that the G2E3‐AS1/let‐7a‐5p and CDKN2B‐AS1/let‐7b‐5p pathways may act as upstream lncRNAs and miRNAs of SLC31A1 to up‐regulate the SLC31A1 expression in breast cancer [11].

SLC31A1 has great potential to regulate immunotherapy and chemotherapy resistance in breast cancer. The infiltration levels of immune cells were gradually growing with the increase of the copy number of SLC31A1, encompassing CD8^+^ T cell, CD4^+^ T cell, B cell, NK cells, macrophage cell, neutrophil cell, and dendritic cell [11, 12]. However, other studies have shown that there exists a negative correlation between the levels of infiltration of CD8 T cells, regulatory T cells, resting mast cells, memory B cells, plasma cells, and activated NK cells and the expression of SLC31A1 [12]. The second study supported that up‐regulation of SLC31A1 could increase the CD4+/CD8+ ratio. Patients with low SLC31A1 expression showed better anti‐CTLA4 treatment effects [12]. Studies have shown that high expression of SLC31A1 is negatively associated with sensitivity to several breast cancer chemotherapy protocols, including CEF (cyclophosphamide, epirubicin, and fluorouracil) plus docetaxel or paclitaxel and AT (anthracycline, taxane) [27]; however, another study has shown that high expression of SLC31A1 in breast cancer predicts that patients are more sensitive to paclitaxel regimens‐alone [12].

SLC31A1 can be regulated by AMPK signal and can also regulate AKT signal and EMT gene‐phenotype to promote the growth of breast cancer. As the upstream of SLC31A1, AMPK and its agonist metformin can significantly improve SLC31A1 level by influencing Nedd4l interaction, which can be used in conjunction with metformin and copper chelating agent for breast cancer treatment [13]. Copper enhances tumorigenesis by activating the PI3K‐PDK1‐AKT signaling pathway in a SLC31A1‐dependent manner, and Nedd4L‐mediated ubiquitination as an upstream negative regulator of SLC31A1 acts as a tumor suppressant by inhibiting the SLC31A1‐PDK1‐AKT oncogenic pathway [9]. In an experiment targeting human breast cancer cells MCF‐7, SLC31A1 silencing significantly eliminated CoCl_2_‐induced E‐cadherin downregulation and vimentin up‐regulation, which inhibited tumor progression by inhibiting CoCl_2_‐induced cytoskeleton rearrangement and EMT marker genes expression [8].

Overexpression of SLC31A1 can significantly enhance the anticancer effect of copper or copper complex [CuCl_2_ (impy)] as a breast cancer therapeutic agent and inhibit breast cancer survival [103]. Pomegranate juice anthocyanidins may induce DNA damage and death of human breast cancer cells MDA‐MB‐231 by up‐regulating SLC31A1 to mobilize intracellular copper ions and ROS production [14].

### 4.2 DMT1

High expression of DMT1 in breast cancer is significantly linked with unfavorable prognosis [15]. As mentioned above, copper uptake by DMT1 requires certain conditions [2]. However, the current research on DMT1 and breast cancer is mostly limited to the function of DMT1 on iron uptake. The involvement of DMT1 in the process of iron uptake was not observed in the untreated TNBC cell line MDA‐MB‐231. However, in iron‐deficient MDA‐MB‐231 cells treated with deferoxamine (DFO), DFO activated the IL‐6/PI3K/AKT signaling pathway and up‐regulated the expression of transferrin Receptor 1 and DMT1. Consequently, this leads to an elevation in the internalization of iron within cells and the facilitation of cellular migration [16]. In human breast cancer biopsies, high expression of HO‐1 was significantly correlated with low expression of DMT1. Further experiments demonstrated that HO‐1 destroyed iron metabolism in breast cancer cells, down‐regulated DMT1, and thus killed breast cancer cells [17]. DMT1 is involved in the regulation of iron metabolism by the Hipo‐Yap pathway in breast cancer [15]. Activated YAP enhances intracellular iron levels by promoting DMT1 and TFR1 expression [15]. Moreover, DMT1 is a direct transcription target of YAP, and its promoter YAP/TEAD‐binding site (CATTCT) can be directly bound by YAP [15].

DMT1 also has an essential function in ferroptosis from breast cancer. Sulfasalazine and DOX (Doxorubicin) not only activate ferroptosis in breast cancer cells by inhibiting xCT and GPX4 but also regulate ferroptosis by activating iron metabolism by up‐regulating DMT1, transferrin, and transferrin receptors [18, 19].

### 4.3 STEAP

STEAP proteins include STEAP1‐4 and atypical STEAP1B; among them, STEAP1‐4 is lower expressed in breast cancer compared with normal tissues, and some people believe that STEAP1‐4 is a tumor suppressor of breast cancer [20, 21, 104]. In breast cancer, STEAP1 and STEAP2 inhibit EMT by inhibiting EMT‐related genes and the PI3K/AKT/mTOR signaling pathway to impede cell proliferation, invasion, and metastasis [20, 21].

### 4.4 ATP7A/B

High expression of ATP7A in breast cancer was significantly linked with reduced survival, while ATP7B showed no statistical difference [22, 23]. ATP7B contributes to the development of breast cancer by participating in the cell cycle, oxidative phosphorylation, and DNA replication pathways [100]. ATP7A silencing inhibits LOX activity in 4T1 breast cancer cell lines in an orthotopic mouse model of breast cancer, leading to the loss of Lox‐dependent metastasis mechanisms, including focal adhesion kinase phosphorylation and recruitment of bone marrow cells to the lung, thereby inhibiting tumor tumorigenesis and metastasis [22]. Besides the loss of LOX metallization, the accumulation of copper and ROS may also play a role in the inhibitory impact of ATP7A, suppressing tumor growth and metastasis [22]. Analysis of the data found that in 16 tumors, including invasive breast cancer, ATP7A, and ATP7B were negatively associated with macrophages [24]. It was found that ATP7A increased the resistance of breast cancer cells to cisplatin and could be inhibited by miR‐148a‐3p to increase the cytotoxicity of cisplatin [23]. PDA‐DTC/Cu is a nanoparticle that assembles copper ions into polydopamine nanostructure, which can not only induce cuproptosis in breast cancer by increasing intracellular copper ion content, but also inhibit the expression of ATP7A/B, and further promote the occurrence of cuproptosis [25]. PCD@Cu is also a composite nanoparticles prepared from ROS‐responsive prodrugs PEG‐TK‐DOX, GSH‐responsive prodrugs PEG‐DTPA‐SS‐CPT, DOX, camptothecin (CPT), and Cu^2+^. PCD@Cu promotes a significant accumulation of Cu^2+^ in TNBC by inhibiting the expression of ATP7B, thereby inducing cuproptosis [26].

### 4.5 ATOX1

The expression of the copper chaperone ATOX1 is significantly upregulated in breast cancer cells. Elevated ATOX1 expression has been found to be correlated with unfavorable prognosis among individuals diagnosed with early‐stage breast cancer [27]. The ATOX1 is observed to accumulate at the borders of lamellipodia in migrating breast cancer cells. It plays a role in facilitating breast cancer cell migration through the coordinated transport of copper in the ATP7A‐LOX axis [28]. DCAC50, a small molecule inhibitor of ATOX1 and CCS, decreases cell proliferation, inhibits angiogenesis, increases copper levels, and increases oxidative stress, thereby triggering apoptosis in TNBC cells and enhancing the cytotoxicity of paclitaxel [29]. CPEB4 and the copper chelators Memo1 and tetrathiomolybdate(TM) can interact with ATOX1 and exchange Cu(I) in vitro, thereby preventing copper‐mediated redox activity [72, 105, 106, 107].

### 4.6 CCS

The Copper Chaperone for Superoxide Dismutase (CCS) is responsible for the regulation of copper transportation to SOD1 within the cytoplasm and mitochondria [71], which exhibits a highly significant level of expression within breast cancer tissues and plays a role in facilitating the proliferation and migration of cancer cells [30, 108]. In studies of MDA‐MB‐231 cells (TNBC) and MCF‐7 cells (ER + BC), exogenous inhibitors DC_AC50 of CCS and endogenous silencing of CCS both inhibit the growth and migration of breast cancer cells by inhibiting the ROS‐mediated MAPK/ERK pathway activity [30].

### 4.7 COX17/CSO1/SCO2/COX11

The mitochondrial Cu chaperone of CCO, COX17, is involved in transporting Cu from the cytoplasm to the mitochondria. High levels of mitochondria‐specific copper chaperone COX17 effectively predict poor prognosis, distant metastasis, recurrence, and tamoxifen‐resistance in breast cancer patients [31]. COX17 deletion limits mitochondrial copper deficiency and inhibition of TM on invasion and metastasis of breast cancer cells [44].

Mitochondrial copper chaperones cytochrome c oxidase 1/2/11(SCO1, SCO2, and COX11) are thought to play roles in cellular copper homeostasis, mitochondrial redox signaling, or the supply of copper ions to mitochondrially encoded cytochrome c oxidase l/II (MTCO1/2, COX1/2) [78, 109]. SCO2 functions as a thiol‐disulfide oxidoreductase that plays a crucial function in regulating the redox state of cysteine residues in SCO1 during the maturation process of MTCO2. One study suggested that miR‐663 reduced the expression of almost all OXPHOS assembly factors in breast cancer cells, including SCO1, and regulated the retrograde signal transduction from mitochondria to the nucleus, thus inhibiting the occurrence of breast cancer [32]. The protein p53 has the ability to regulate metabolic pathways in human breast cancer through its interaction with the protein SCO2 [33]. Hepatitis B X‐interacting protein (HBXIP), an oncoprotein, suppresses PDHA1 and SCO2 in breast cancer to improve glucose metabolism reprogramming [34]. COX11 mainly transfers copper ions to MTCO1 [3]. Data analysis of STXBP4/COX11 polymorphisms and breast cancer risk showed that alleles of COX11 (rs17817901, rs6504950) were positively associated with breast cancer risk [35, 36].

### 4.8 MTs/GSH

Just like iron storage proteins, MT1/2 binds to multiple copper ions and is responsible for copper storage [1]. The regulation of MT1 and MT2 on breast cancer cells is mainly reflected in promoting the cell cycle [37], inhibiting apoptosis [38], enhancing invasiveness [39], regulating oxidative stress [40], and increasing chemotherapy resistance [41]. Experiments have shown that activation of p53 is an important factor in the metal regulation of MTs, and only p53 intact breast cancer epithelial cells can induce the synthesis of MTs after metal exposure [110].

MT1 and MT2 in ductal breast cancer were positively linked with Ki‐67 and MCM‐2 antigen expression and negatively correlated with estrogen receptors (ER) and progesterone receptors (PR) expression but had no relationship with HER‐2 expression [111, 112]. MT‐2A may promote the progression of the cell cycle from the G1 phase to the S phase through the ATM/Chk2/cdc25A pathway [37]. The upregulation of MMP‐9 through the activation of AP‐1 and NF‐κB is facilitated by MT‐2A, thereby promoting the invasion and migration of breast cancer cells [39]. MT1/2 is involved in regulating oxidation status in DOX‐treated MCF‐7 cells [40]. Down‐regulation of MTs in MCF‐7 cells by silencing the MT‐2A gene increased the chemosensitivity of the cells to DOX [41]. Melatonin and arsenic may enhance the proliferation of breast cancer cells by up‐regulating MT1/2 [87, 113].

GSH is known to be involved in antioxidant defense and signal transduction against ROS, regulating redox homeostasis in breast cancer cells [43]. GSH also connects the uptake and transport of copper and stores copper ions [85]. Based on its basic function, GSH acts as a copper chelator to block the copper accumulation in cells. Recent studies have shown that GSH blocks ES‐induced cuproptosis by chelating intracellular copper ions and that buthionine sulfoximine increases the tumor cells' sensitivity to cuproptosis by consuming GSH [4]. Theoretically, p53 may regulate cuproptosis by inhibiting GSH production [114]. Cuproptosis has been confirmed in breast cancer cells, and copper overload caused by GSH consumption after breast cancer treatment with type‐I AIE photosensitizer‐loaded biomimetic system (PTC) in vivo and in vitro results in the aggregation of lipoylated proteins and the loss of iron–sulfur proteins, ultimately culminating in cuproptosis [42].

## 5 Conclusions and Future Prospects

Common copper transport system members include copper inflow proteins (SLC31A1, DMT1), copper outflow proteins (ATP7A, ATP7B), copper chaperones (ATOX1, CCS, COX17, etc.), and copper storage proteins (MTs, GSH). Members of the copper transport systems maintain high expression in breast cancer [10, 15, 27, 30, 31, 33, 58, 100, 101] and can lead to poor prognosis of breast cancer by maintaining and promoting copper homeostasis, redox state, occurrence and development, invasion and metastasis, chemotherapy resistance, and other aspects. The unstable expression of its members may also lead to the growth or death of breast cancer due to the double action of copper and other factors.

In addition, SLC31A1 promotes the development of breast cancer by activating the PI3K‐PDK1‐AKT signaling pathway and the EMT (Epithelial–mesenchymal transition) gene‐phenotype [9]. The ATOX1‐ATP7A‐LOX axis may promote the migration of breast cancer cells by promoting angiogenesis [28]. CCS promotes the growth and migration of breast cancer cells by regulating ROS‐mediated ERK1/2 activity [30]. MTs can activate AP‐1 and NF‐κB, thereby up‐regulating MMP‐9 and enhancing invasiveness, thus promoting the proliferation of breast cancer cells [39]. Remarkably, depletion of GSH (Glutathione) induced copper overload ultimately leads to cuproptosis in breast cancer cells by leading to lipoacylated protein aggregation and iron–sulfur protein loss [42]. Overactivation of SLC31A1 can also induce DNA damage and death in breast cancer cells by increasing intracellular copper ions and ROS [14].

Copper depletion and overload are both good treatments for breast cancer. Copper chelating agent tetrathiomolybdate reduces TNBC transfer by decreasing mitochondrial oxidative phosphorylation [44]. Hydroxytyrosol down‐regulates the phosphorylation of copper‐dependent AKT by reducing intracellular copper levels, thereby inhibiting TNBC invasion [45]. Triethylenetetramine inhibits AKT‐driven EMT activation by reducing the bioavailability of copper in breast cancer cells [46]. Nanomedicines such as Typei AIE photosensitizer‐loaded biomimetic system, PDA‐DTC/Cu and PCD@Cu, as well as the compound Pyrithione zinc, have been demonstrated to induce cuproptosis in breast cancer cells by disrupting copper homeostasis, depleting glutathione, and oligomerizing DLAT [25, 26, 42, 48].

In conclusion, the functions of members of the copper transport system in tumors are diverse, but their roles and mechanisms in breast cancer have not been studied much and have limitations in preclinical studies. Targeting members of the copper transport system through the regulation of copper homeostasis or other functions in the treatment of breast cancer is a challenging and promising strategy.

## Author Contributions

**Yichang Chen:** investigation (equal), project administration (equal), writing – original draft (lead). **Chen Li:** data curation (equal), writing – original draft (equal). **Mengxin Li:** project administration (equal). **Bing Han:** investigation (equal), project administration (equal).

## Ethics Statement

The authors have nothing to report.

## Conflicts of Interest

The authors declare no conflicts of interest.

## Data Availability

The authors have nothing to report.
